# Persistent immune thrombocytopaenic purpura associated with SARS‐CoV‐2 infection

**DOI:** 10.1002/jha2.201

**Published:** 2021-05-04

**Authors:** Yoshiki Furukawa, Miki Ando, Yoko Azusawa, Shintaro Kinoshita, Sakiko Harada, Tomonori Ochiai, Tadahiro Honda, Kazuya Sugimoto, Yoko Tabe, Norio Komatsu, Jun Ando

**Affiliations:** ^1^ Department of Hematology, Juntendo University School of Medicine Tokyo Japan; ^2^ Department of Transfusion Medicine and Stem Cell Regulation, Juntendo University School of Medicine Tokyo Japan; ^3^ Department of Next Generation Hematological Laboratory Medicine Juntendo University School of Medicine Tokyo Japan

We describe evolution of persistent immune thrombocytopaenic purpura (ITP) from acute ITP in a young woman with clinically otherwise inapparent severe acute respiratory syndrome ‐ coronavirus 2 (SARS‐CoV‐2) infection (COVID‐19); her development of ITP was matter for an earlier report.[1] ITP is an acquired disease in which thrombocytopaenia results from autoantibodies against platelet antigens. Approximately 10% of patients with acute ITP develop persistent (lasting 3–12 months) or chronic (>12 months) ITP.[2] Infections with viruses like Epstein‐Barr virus and cytomegalovirus can trigger acute ITP.[3] Many instances of acute ITP associated with COVID‐19 are described [1, 4, 5, 6]. Instances of persistent or chronic ITP associated with COVID‐19 have not been reported.

A previously well 30‐year‐old woman sought dental care for new‐onset gingival bleeding in August 2020. A markedly decreased platelet count (3 × 10^9^/L) was found. She was referred to our hospital.

On admission, her gums bore coagulated blood and her extremities prominent purpural and petechial lesions (Figure 1A). She denied medication. A platelet count was very low (4 × 10^9^/L; 2018, 164 × 10^9^/L) with a high increase in reticulated platelet count (28.6%, expected < 2.0%). Platelet‐associated IgG was elevated at 559 ng/10^7^ platelets (expected < 46 ng/10^7^). No other haematologic abnormality was found in peripheral blood. Current infection with Epstein‐Barr virus, cytomegalovirus, hepatitis B virus, hepatitis C virus, or human immunodeficiency virus ‐1 and ‐2, autoantibodies (Table 1), and *Helicobacter pylori* antigen (tested in stool) were not detectable. Bone‐marrow examination showed markedly increased numbers of megakaryocytes (264 × 10^6^/L), without dysplasia (Figure 1B). Computed tomography identified no splenomegaly and unexpectedly found bilateral ground‐glass lower‐lobe lung opacity; the patient was afebrile, and chest roentgenograms had been assessed as without abnormality. Real‐time polymerase chain reaction (RT‐PCR) testing for SARS‐CoV‐2, using saliva, accordingly was performed 2 days in a row, in both without detecting evidence of infection.

**FIGURE 1:**
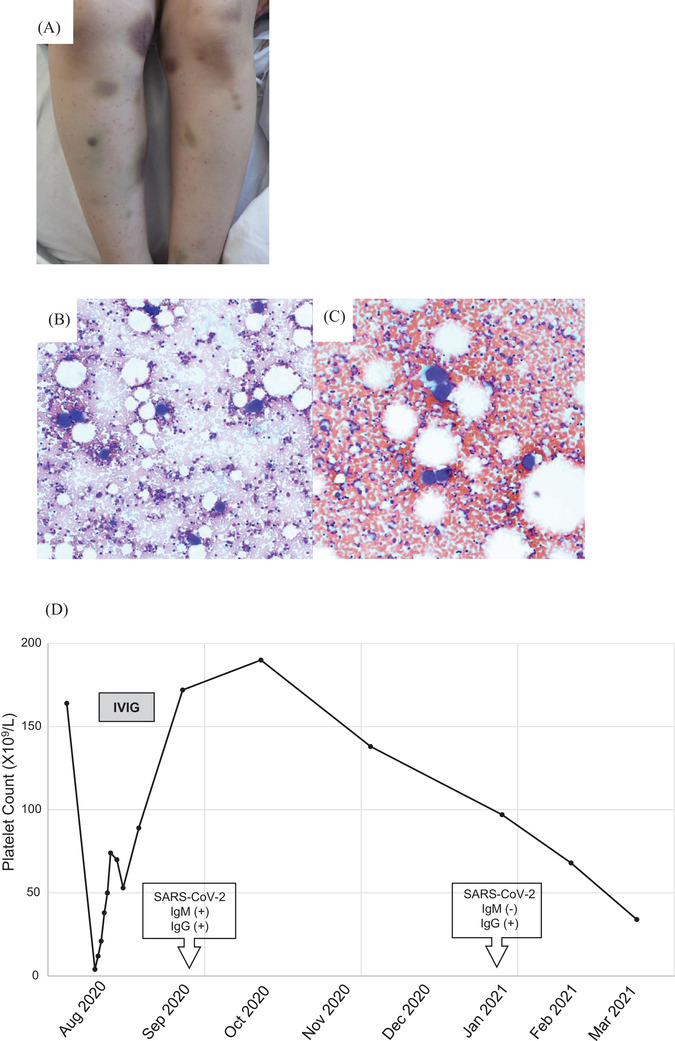
(A) Purpuric lesions and petechiae, both legs, at presentation. (B and C) Normocellular bone‐marrow aspirate with increase in megakaryocyte numbers (May‐Grünwald‐Giemsa, original magnifications, 100 x); (B) August 2020 and (C) March 2021. (D) Course, platelet count Abbreviations: IVIG, intravenous immunoglobulin therapy; SARS‐CoV‐2, severe acute respiratory syndrome ‐ coronavirus 2; SARS‐CoV‐2 IgG, anti‐SARS‐CoV‐2 IgG‐class antibody; SARS‐CoV‐2 IgM, anti‐SARS‐CoV‐2 IgM‐class antibody

**TABLE 1:** Results, assays for infection and autoantibodies

	August 2020	March 2021
**Agent**		
HIV	(‐)	(‐)
Hepatitis B virus	(‐)	(‐)
Hepatitis C virus	(‐)	(‐)
Epstein‐Barr virus	IgG: 160x, IgM < 10x, EBNA 160x	IgG: 80x, IgM < 10x, EBNA 20x
Cytomegalovirus	(‐)	(‐)
*Helicobacter pylori*	(‐)	Not assayed
**Autoantibodies**		
Anti‐nuclear	(‐)	(‐)
Anti‐SS‐A	(‐)	(‐)
Anti‐SS‐B	(‐)	(‐)
Anti‐DNA (IU/ml)	<2.0	<2.0
Anti‐platelet	(‐)	(‐)
Anti‐β2‐glycoprotein I (U/ml)	<1.2	<1.2

Abbreviations: Anti SS‐A, anti‐Sjögren's‐syndrome‐related antigen A autoantibodies; Anti‐SS‐B, anti‐Sjögren's‐syndrome‐related antigen B autoantibodies; HIV, human immunodeficiency virus ‐1 and ‐2; EBNA, Epstein‐Barr virus nuclear antigen.

Acute ITP was diagnosed, and intravenous immunoglobulin therapy (IVIG), 400 mg/kg, was initiated. Corticosteroids were not used because of possible pneumonia. On the fourth day of IVIG, her platelet count had increased to 38 × 10^9^/L. Nasopharyngeal RT‐PCR testing detected SARS‐CoV‐2 sequences. Haemorrhagic disease responded to IVIG. The patient was discharged home on hospital day 10 [1].

She attended clinic monthly in follow‐up. In October 2020, her platelet count had recovered (190 × 10^9^/L); however, it gradually fell again (97 × 10^9^, 68 × 10^9^, 30 × 10^9^/L: January, February, March 2021, respectively). Anti‐SARS‐CoV‐2 immunoassays of serum detected anti‐SARS‐CoV‐2 IgG‐ but not IgM‐class antibody. Autoantibodies and evidence of infection again were absent (Table 1), and bone‐marrow examination findings were like those before (increase in megakaryocyte numbers at 51 × 10^6^/L without dysplasia [Figure 1C]). Persistent ITP following acute ITP associated with COVID‐19 was diagnosed. As her platelet count is steady at >30 × 10^9^/L (Figure 1D) without evident bleeding, corticosteroid therapy has been withheld in favour of close observation.

Mild thrombocytopaenia has been observed in approximately 5%–10% of patients with COVID‐19 infection [7]. Implicated factors and mechanisms include cytokine storm, direct infection of haematopoietic and bone‐marrow stromal cells, antibody‐mediated platelet destruction, reduced effect of thrombopoietin, increased circulating‐platelet consumption via lung injury or multiple‐organ failure, and immune complexes [8, 9]. Recent guidelines recommend steroids as first‐line therapy for ITP associated with COVID‐19. IVIG is recommended when an immediate rise in platelet count is required [10]. Administration of thrombopoietin‐receptor agonists may be associated with increased risk of thrombosis and hepatobiliary biomarker abnormalities; that these agents be avoided in COVID‐19 is recommended at present [5, 10]. As noted above, we refrained from administering corticosteroids for fear of potentiating infection via immunosuppression. Platelet counts promptly rose with IVIG treatment.

Failure to develop anti‐SARS‐CoV‐2 antibodies associated with immunochemotherapy (e.g., rituximab, corticosteroids) is described [11, 12]. Our patient, by contrast, had been well, receiving no such treatment. Anti‐SARS‐CoV‐2 antibodies of both IgM‐ and IgG‐class were present on initial presentation, and on repeat evaluation prompted by recurrence of thrombocytopaenia only IgG‐class anti‐SARS‐CoV‐2 antibody was demonstrable, consonant with remote COVID‐19 and persistent thrombocytopaenia. Bennett et al describe relapse of COVID‐19‐associated thrombocytopaenia 28 days after response to IVIG therapy and propose pre‐existent and undiagnosed ITP [13]. In our patient, a previously normal platelet count may argue against that hypothesis.

Mahevas et al describe 14 patients with COVID‐19‐associated ITP, of whom three relapsed (with recovery) on day 30, day 35, and day 58 during follow‐up [5]. As their follow‐up period was only 50–60 days, whether persistent or chronic ITP developed is unknown. The authors point out, however, that COVID‐19 may trigger a tolerance breakdown that could lead to persistent or chronic ITP.

We believe that our patient is the first instance described of persistent ITP (>7 months follow‐up) evolved from acute ITP associated with COVID‐19. Thrombocytopaenia is a common comorbidity in COVID‐19, likely with several aetiologies, among them ITP. As acute ITP triggered by events other than COVID‐19 may progress to persistent or chronic ITP even after initial recovery in platelet count, we anticipate that more patients like ours will be encountered. Continuous and long‐term monitoring of acute ITP associated with COVID‐19 thus must be considered essential.

## CONFLICT OF INTEREST

The authors declare that there was no conflict of interest in carrying out this study.

## AUTHOR CONTRIBUTIONS

Yoshiki Furukawa and Yoko Azusawa wrote the manuscript. Miki Ando directed the study and wrote the manuscript. Jun Ando designed the project. Yoko Tabe performed anti‐SARS‐CoV‐2 immunoassays. Shintaro Kinoshita interpreted data. Sakiko Harada, Tomonori Ochiai, Kazuya Sugimoto, Tadahiro Honda and Norio Komatsu reviewed the manuscript and provided scientific discussions. All authors have read and approved the manuscript.
